# Performing the HINTS-exam using a mixed-reality head-mounted display in patients with acute vestibular syndrome: a feasibility study

**DOI:** 10.3389/fneur.2025.1576959

**Published:** 2025-05-14

**Authors:** Nadia Sadok, Gijs Luijten, Fin H. Bahnsen, Christina Gsaxner, Lorenz Peters, Theda Eichler, Theresa Rombach, Stephan Lang, Sameh Khattab, Jens Kleesiek, Dagny Holle, Moritz Meyer, Jan Egger

**Affiliations:** ^1^Department for Oto-Rhino-Laryngology, Head and Neck Surgery, Essen University Hospital (AöR), University Essen-Duisburg, Essen, Germany; ^2^Institute for Artificial Intelligence in Medicine (IKIM), Essen University Hospital (AöR), Essen, Germany; ^3^Institute of Computer Graphics and Vision (ICG), Graz University of Technology, Graz, Austria; ^4^Center for Virtual and Extended Reality in Medicine (ZvRM), University Hospital Essen, Essen, Germany; ^5^Institute for Medical Education, Center for Translational Neuro- and Behavioral Sciences (C-TNBS), University Hospital, University of Duisburg-Essen, Essen, Germany; ^6^Cancer Research Center Cologne Essen (CCCE), West German Cancer Center Essen, University Hospital Essen (AöR), Essen, Germany; ^7^German Cancer Consortium (DKTK), Essen, Germany; ^8^Department of Physics, TU Dortmund University, Dortmund, Germany; ^9^Faculty of Computer Science, University of Duisburg-Essen, Essen, Germany; ^10^Department of Neurology, West German Headache and Vertigo Center, Essen University Hospital (AöR), Essen, Germany

**Keywords:** acute vestibular syndrome, mixed reality headset, head-mounted display, eye movement, nystagmus, vertigo, feasibility study, head impulse-nystagmus-test of skew

## Abstract

**Background:**

In patients with acute vestibular syndrome (AVS) differentiating between benign acute peripheral vestibular disorders and possible life-threatening central, causes such as stroke, can be challenging due to similar symptoms. AVS patients experience dizziness, vertigo, imbalance, nausea, vomiting, and abnormal eye movements. This research evaluates the feasibility of using the eye-tracking capability of a mixed reality optical-see-through head-mounted display (MR-OST-HMD) to detect pathological eye movement patterns in patients with AVS.

**Methods:**

Conducted at University Hospital Essen, this study assessed patients with AVS using a MR-OST-HMD during the HINTS-Exam. The feasibility study included 21 healthy subjects, seven patients with acute peripheral vestibular dysfunction and two stroke patients. Eye gaze, head position, and orientation were captured using a MR-OST-HMD and an in-house developed application designed to simulate the HINTS-Exam. The eye-tracking technology determined gaze direction and position, while the internal measurement unit and gyroscope recorded head movements in terms of position and velocity.

**Results:**

The MR-OST-HMD detected abnormal eye movements, including nystagmus, saccades, and skew deviation effectively. The device proved effective even for patients with severe nausea and elderly participants, who completed the eye calibration and HINTS-Exam without difficulty. The MR-OST-HMD HINTS-Exam was quick to perform (approximately 5 min) and was easily integrated into clinical practice after a single demonstration for medical staff.

**Conclusion:**

MR-OST-HMD can detect pathological eye movements in AVS patients. Future research should validate these findings in larger cohorts and explore machine learning integration to enhance diagnostic accuracy.

## 1 Introduction

In patients with acute vestibular syndrome (AVS), differentiating benign acute peripheral vestibular disorders, such as vestibular neuritis, from potentially life-threatening central causes, such as posterior circulation stroke, can be challenging due to the similarity of symptoms. Patients with AVS suffer from acute dizziness or vertigo, imbalance, nausea, vomiting, and abnormal eye movements known as nystagmus, with symptoms considered acute within the first 72 h after onset (1). Approximately 10–20% of patients presenting to the emergency department with dizziness have an AVS (2). In the United States, vertigo and dizziness are accountable for 4.4 million visits to the emergency department annually, with vestibular disorders accounting for up to 33% (3). The cost per patient associated with vertigo is between 3341 and 5470 GBP in the UK and 13.717 USD in the United States (4). These figures do not include posterior circulation strokes, which significantly affects the patient’s quality of life. 10–33% of AVS cases are attributed to posterior circulation stroke (5–10). Only 20% of these stroke cases exhibit focal neurologic signs, while the majority manifest isolated AVS (1–4, 11). This presents a challenge in the early diagnosis of stroke, as it is believed that up to 35% of strokes go undetected (12, 13).

The HINTS-Exam (Head-Impulse, Nystagmus, Test of Skew) is a clinical examination conducted on patients presenting with AVS (1–4, 11–15). It is the gold standard for differentiating peripheral vestibular dysfunction from central causes. The HINTS-Exam has shown to be more sensitive than neurological imaging techniques such as computed tomography (CT) imaging and diffusion-weighted magnetic resonance imaging (DW-MRI) in excluding a stroke in patients with AVS (1, 11). The HINTS-Exam is a bedside test performed in a few minutes without additional equipment. An abnormal head impulse test, for example, showing VOR dysfunction with unilateral nystagmus and normal skew deviation, suggests a peripheral cause. In contrast, a normal head impulse test, with direction-changing, vertical nystagmus or a skew deviation indicates a central cause and necessitates immediate further investigation (1–4, 11, 14–16). A disadvantage of the HINTS-Exam is that it requires expertise and practice. Consequently, neurologists and otorhinolaryngologists primarily conduct the HINTS-Exam. However, other health care professionals lacking this specific expertise and practice (11, 17) often initially triage patients presenting with an AVS. This can result in false referrals and delayed treatment for stroke patients (11, 17). Other diagnostic instruments are available to measure and assess the vestibular organs, such as Videonystagmography (VNG), caloric reflex testing, and the Video-Head-Impulse-Test (vHIT) (18, 19). These tools evaluate the vestibular system’s function and are highly valuable for diagnosing vestibular dysfunctions. However, these devices are not universally available and especially not available in every emergency department. They are expensive, approximately 10,000–15,000 euros, and are generally not portable. However, in the case of ischemic stroke, swift diagnosis and initiation of treatment are essential to limit long-term brain damage (20).

This research aimed to provide a mobile and cost-effective tool that improves and optimizes the triage of patients with AVS, thus helping to avoid delays in the diagnosis and treatment of stroke patients. We assessed whether a mixed reality optical-see-through head-mounted display (MR-OST-HMD) is capable of detecting pathological eye movement patterns in patients experiencing acute vestibular syndrome.

## 2 Methods

### 2.1 Study design

This feasibility study was designed to explore the potential of the HoloLens 2 (HL2) (Microsoft Corp., Redmond, Washington, United States), a MR-OST-HMD, to distinguish between patients experiencing acute vertigo and healthy individuals by measuring and plotting (pathological) eye movements during the HINTS-Exam. The Microsoft HoloLens 2 used within the study has optical-see-through glasses, sensors, and an on-board computer.

### 2.2 Ethical considerations

The medical ethical committee (23-11518-BO) approved the study. As this is a feasibility study, it does not meet the ICMJE criteria for trial registration.

### 2.3 Participants

Patients presenting with symptoms of AVS at the department of neurology, department of otorhinolaryngology, or the emergency department of the university hospital Essen, Germany were included in this study. Inclusion criteria were patients experiencing acute dizziness or vertigo combined with imbalance, nausea, or vomiting. As a control group, individuals without vertigo, neurological, or vestibular pathologies were included. All participants are >18 years. Exclusion criteria were a history of neurological disorders or previous vestibular dysfunction.

### 2.4 Procedures

Upon admission, eligible participants underwent a standard clinical assessment, including the clinical HINTS-Exam, conducted by trained neurologists and/or otorhinolaryngologists. Subsequently, participants underwent additional diagnostic procedures, including VNG and/or the vHIT, to confirm the diagnosis and cause of the AVS. CT imaging or DW-MRI was performed in cases with suspected stroke.

Immediately following the clinical HINTS-Exam, the in-house developed application installed on the HL2 was performed. The application captured left-, right- and combined- eye gaze and the wearer’s head position and orientation. Before starting the application individual eye distance and position was calibrated thereby ensuring accurate tracking and comparable data acquisition. Only a brief demonstration followed by a single supervised exercise was provided to the medical team before using the in-house developed application on the HL2 in a clinical setting.

### 2.5 Microsoft HoloLens 2

The HL2 is an advanced MR-OST-HMD that incorporates four visible light cameras and a depth (1-Mega Pixel time-of-flight) sensor enabling spatial mapping via simultaneous localization and mapping algorithm (SLAM). The internal measurement unit (IMU), composed of an accelerometer, gyroscope, and magnetometer, enables the HL2 to update its position within the spatial map accurately and continuously via a Kalman filter method using SLAM and the IMU’s measurements as input. Its two infrared eye-tracking cameras and IMU measure head position and gaze. The HL2 uses advanced eye-tracking technology to detect the user’s gaze precisely. This technology involves projecting near infrared patterns onto the eyes and using cameras to track the reflections from the cornea and the pupil. This allows the system to determine the position of the pupil and the gaze vector per eye. Besides that, the cameras are used for hand tracking and gesture recognition. The gyroscope is crucial for determining the device’s orientation by measuring the angular velocity around the device’s axis. The gyroscope contributes to the headset’s ability to understand and track the user’s head movements in real-time. Tracking the user’s head position, orientation, and eye gaze within the virtual spatial map of the real world ensures that the virtually augmented content stays anchored correctly in the real world, regardless of the user’s movements, by updating the virtual images within the optical-see-through glass. In contrast to numerous other MR headsets, the optical-see-through glasses permit the user to perceive the world as if they were looking through conventional glasses, in contrast to video-see-through where the real world is filmed by cameras and projected onto a screen.

### 2.6 Relevant features: the MR-OST-HMD HINTS exam application

To gather the necessary data about from the HL2, an in-house application was developed that simulated the HINTS-Exam. The application uses Unity3D (Unity Technologies, San Francisco, California, United States), the Mixed Reality Toolkit 3 (MRTK3) and Extended Eye Tracking SDK (21, 22). The application uses only open-source software tools. It can be readily deployed on devices with eye-tracking capabilities if the proprietary company is involved in MRTK3, such as the Meta Quest Pro (Meta Platforms, Inc., Menlo Park, California, United States).

**Figure 1:**
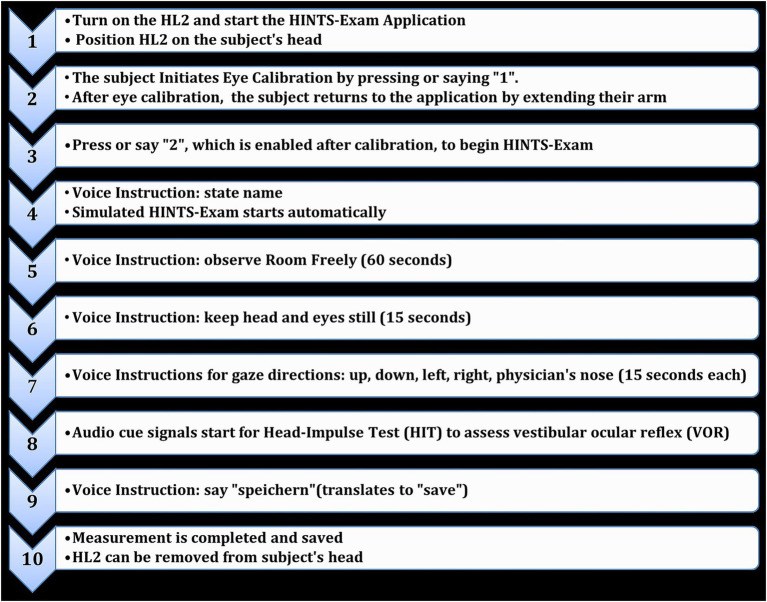
The ten steps to perform the HINTS exam using the HoloLens 2 HINTS application and save the measured data.

**Figure 2:**
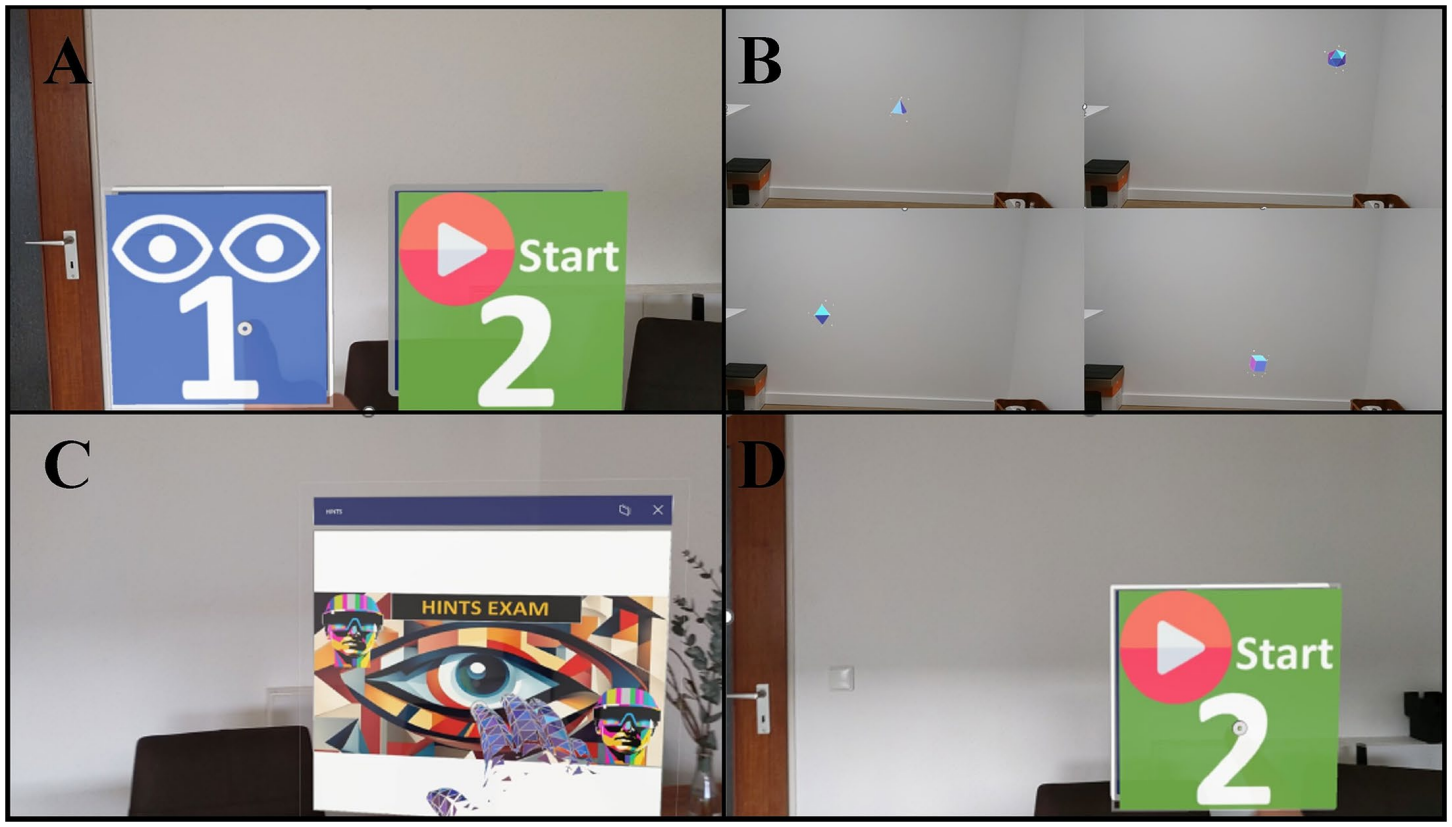
The subject receives the HL2 and will always see the two options aligned with their line of sight **(A)**, the calibration is started by pressing or saying the number one. The subject is instructed to follow the virtual cubes as part of Microsoft HL2’s proprietary eye calibration **(B)**. The application window is in the same position as at the start of the calibration and can be activated by extending the arm **(C)**. The user can press or say the number two to start the application **(D)**. The user is then guided through the HINTS test with pre-recorded voice messages and the user data is automatically saved at the end, but can also be forced by voice command.

The simulated HINTS-Exam is comprised of ten steps (see Figures 1, 2). The measured data obtained with the application include a timestamp, the position in cartesian coordinates, and gaze direction as a unit vector of the left and right eye and eyes combined in relation to the world or eye-tracking cameras. Additionally, the position and orientation of the head within the spatial map are determined by the HL2. The orientation is expressed in Euler angles and quaternions. The HL2 establishes the origin of the (virtual) world at the startup position, rendering it more reliable for comparing positional differences over time or in relation to the eye-tracking cameras than the exact values themselves. These are referred to as features per frame, which will subsequently be employed to illustrate nystagmus, pathologic corrective saccades because of a decreased angular VOR, and skew deviation.

The eye-tracking sensor recorded measurements at 60 Hertz (Hz) (22), with one measurement per frame. IMU sensors operate at hundreds of Hz, but head-related measurements, as processed by the HL2’s proprietary algorithms during runtime, were also recorded once per frame, ensuring consistent timestamps across all measurements.

The post-processing of the raw data generates comma-separated values for each subject and each part of the HINTS exam, along with a label indicating the patient’s diagnosis. Every recorded frame has a timestamp and accompanying measurements. Post-processing and feature extraction was conducted using Python 3.11 (23), mainly using Pandas version 2.1.4 (24), and Plotly version 5.20.0 (25).

#### 2.6.1 Head-Impulse-Test

Pathologic corrective saccades because of a decreased angular VOR, henceforth referred to as saccades, were detected by first computing eye velocity and then rotational velocity of the head. The eye velocity is computed based on changes in the horizontal component of the positional vector over time for the left and right eye combined within the world coordinate system. For the rotational velocity of the head, the Euler angle corresponding to rotation around the vertical axis of the head was used. The changes over time were used to compute angular velocity. To facilitate interpretation, both features were centered around the mean. Furthermore, the eye and angular head velocities were multiplied by different factors for ease of interpretation.

#### 2.6.2 Nystagmus

For horizontal and down beating (vertical) nystagmus, the pattern for unhealthy subjects becomes evident when plotting the gaze direction in the horizontal (*x*-axis) or vertical (*y*-axis) of the right and left eye combined over time in relation to the eye tracking camera. The direction is indicated in unit vector form, which means that changes are relatively small but discernible. Again, the values of the left eye gaze direction plus the right eye gaze direction are centered around the mean.

#### 2.6.3 Test of skew

The vertical position of the eye in relation to the eye-tracking camera over time can display the skew deviation. Microsoft mixed reality systems use the right-handed coordinate system. Consequently, the vertical position relates to the *y*-axis information within our post-processed data. The physician’s nose was used as the fixation target at approximately 40 cm, although a point on the wall could also serve this purpose. To ensure diagnostic accuracy, the presence of skew deviation was clinically confirmed using the alternating cover test by a neurologist. A vertical eye movement during alternating occlusion of the eyes was defined as skew deviation.

## 3 Results

### 3.1 Participants

In total, 30 participants were included: 21 healthy subjects, seven patients with acute peripheral vestibular dysfunction and two stroke patients. The mean age of the healthy group was 33 years, and 66% were female. The mean age for the patients with peripheral vestibular dysfunction was 58 years, and 71% were female. Both stroke patients were male, 67 and 80 years old. All patients underwent the standard clinical examinations, including the standard HINTS-Exam, VNG and vHIT, that allow a reliable diagnosis.

### 3.2 Usability of the MR-OST-HMD HINTS-exam application

One patient with a cochlear implant had initial measurement problems due to the positioning of the MR-OST-HMD headband and cochlear implant. After adjustments to the adjustable headband of the MR- OST-HMD, the cochlear implant was compatible, and the patient could hear and follow all of the pre-recorded voice prompts. Most patients were able to follow all instructions immediately. Even in individuals with severe nausea, the eye calibration and examination using the MR-OST-HMD HINTS-Exam application could be completed without interruption. Elderly patients responded intuitively to the HINTS-Exam application, with no issues during the measurements. During the Head-Impulse-Test, as described, the head is abruptly rotated. Concerns arose that the MR-OST-HMD IMU might not accurately detect head movement due to head movements shifting the device’s position. Performing the Head-Impulse-Test from behind by the examiner made for better stabilization of the MR-OST-HMD and induced less involuntary movement of the MR-OST-HMD compared to standing in front of the patient. A black dot on the wall served as the fixation target at approximately 40 centimeters. The performance of the MR-OST-HMD HINTS exam took approximately 5 min, and was considered not time-consuming by the medical staff, especially when compared to vHIT or VNG. After one demonstration, the medical staff could integrate the device effectively in the clinical setting without further assistance.

### 3.3 Data analyses

**Figure 3:**
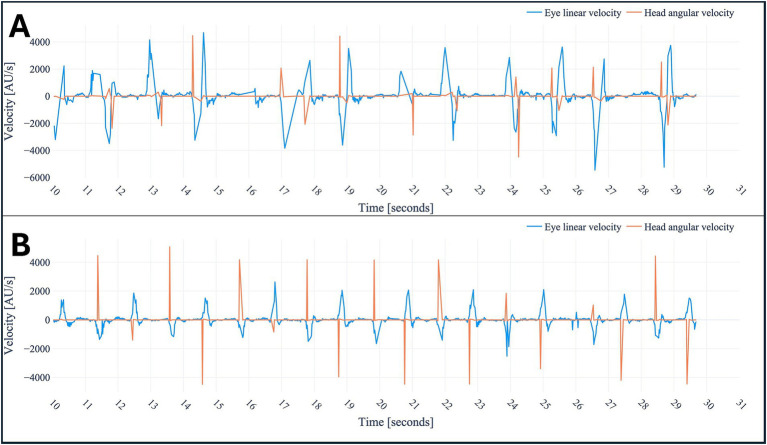
Head-Impulse-Test in a healthy subject **(B)** and patient suffering from saccades in acute peripheral vestibular dysfunction **(A)**, respectively. The combined horizontal linear eye velocity and angular head velocities were multiplied by different factors for ease of interpretation. Due to this multiplication and utilizing different velocity measured the notation arbitrary unit (AU) was used. Peak heights differ due to variations in head velocity (physician-applied force) and eye velocity (subject-dependent), but the overall pattern remains unchanged.

**Figure 4:**
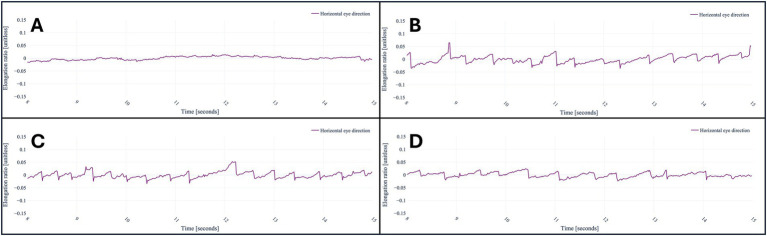
A healthy subject looking at the nose of the physician **(A)**, a patient suffering from acute peripheral vestibular dysfunction looking at the nose of the physician **(B)**, to the left **(C)**, and right **(D)**. The baseline is centered around its mean; thus, the values represent the changes within the x component of the unit vector caused by nystagmus.

**Figure 5:**
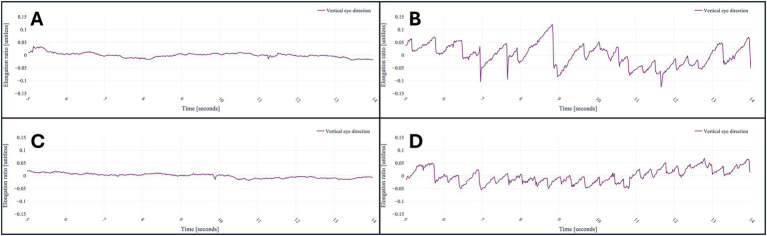
A healthy subject looking straightforward **(A)** and looking at the nose of the physician **(C)**, respectively. A patient with down beating nystagmus on the right. The patient is looking straightforward **(B)** and at the nose of the physician **(D)**, respectively. The baseline is centered around its mean; thus, the values represent the changes within the y component of the unit vector caused by the down beating nystagmus.

**Figure 6:**
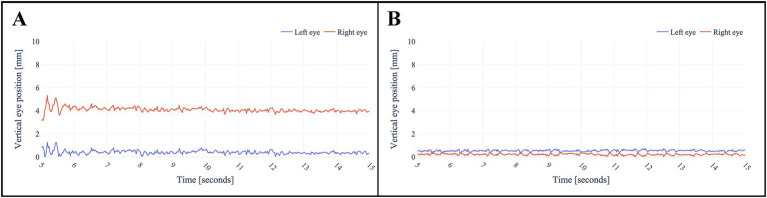
Skew deviation in a stroke patient **(A)** versus a healthy subject **(B)**. Both baselines are centered around zero for a more straightforward interpretation. The physician’s nose served as the fixation target. To ensure diagnostic accuracy, the presence of skew deviation was clinically confirmed using the alternating cover test by a neurologist.

After analyzing the data of the registered eye- and head-movements during the application, saccades, nystagmus and skew deviation were all successfully plotted in graphs (Figures 3–6). Figure 3 demonstrates the visualization of saccades for a patient suffering from acute peripheral vestibular dysfunction. Nystagmus is illustrated in Figures 4, 5. The right side of Figure 6 demonstrates a skew deviation pattern in a stroke patient. As a reference, the results of a healthy subject are displayed on the left side of Figure 6.

Upon initial examination of the data, it was observed that considerable noise and head or eye movement occurred within the first seconds of measurements. This is presumed to be a consequence of the duration of the pre-recorded voice instruction and the subject’s adherence to it. Consequently, the initial seconds were not subjected to analysis.

## 4 Discussion

Rapid differentiation of the cause of dizziness allows early identification of stroke patients. The HINTS-Exam is appropriate in trained hands, but can be misinterpreted by inexperienced personnel. Therefore, the HINTS-Exam is often not used in emergency care due to a lack of knowledge or experience. A machine learning-based tool could increase the number of patients who can be treated in the stroke lysis window, potentially reducing morbidity and mortality. A machine learning-based tool is expected to be especially effective with the MR-OST-HMD approach due to its affordability, portability, and ease of use with minimal training, ideal for emergency or acute out-of-clinic settings. As a first step, this research evaluates the possibility of detecting acute vertigo symptoms using a MR-OST-HMD.

### 4.1 Principal findings

The preliminary results demonstrate that the MR-OST-HMD tested in this study can detect abnormal eye movement patterns, including saccades, various types of nystagmus, and skew deviation. This shows the feasibility of using the HL2 with the in-house developed application to perform the HINTS exam. The MR-OST-HMD HINTS-Exam is straightforward to use for elderly patients within our study and or those wearing hearing aids and does not increase vertigo. It shows that the MR-OST-HMDs can be used as a mobile method for performing the HINTS exam.

### 4.2 Comparison to the literature

MR-OST-HMDs have been implemented across various research studies and have grown in popularity within the medical field (26). Its diagnostic value for patients experiencing dizziness has been explored, showcasing its potential as a tool (27). One study validated the MR-HMD used in this study as an effective instrument for assessing vestibular functions, integrating traditional diagnostic methods with its tracking features. The study demonstrated the device’s accuracy in measuring gait and mobility by conducting Dynamic Gait Index tests on twenty-six healthy adults, suggesting its capacity to enhance vestibular diagnostics. Notably, no patients with acute dizziness were included (28). A systematic review of 141 studies also evaluated the increasing application of eye-tracking technology in optometry. This review unveiled the wide range of uses for eye trackers in diagnosing and managing ocular disorders, particularly emphasizing infrared-based devices and metrics such as fixations and saccades (29). Another study trained a deep-learning model designed to measure nystagmus by identifying ocular regions based on both real and virtual-reality-generated images of eye movements on video. They showed a high accuracy in tracking horizontal and vertical eye movements from video footage. However, detecting torsional eye movements was challenging, and the model did not evaluate saccades and skew deviation (30). One research group explored using an iPhone attached to 3D-printed goggles as a portable VNG tool (31). Another study employed a prototype iPhone app to successfully perform the vHIT on one healthy individual and one patient with acute vestibular neuritis (32). Both studies investigated using portable devices to examine parts of the exam, however, neither focused on the entire HINTS-exam.

### 4.3 Strengths and limitations

This is the first research project to use a MR-OST-HMD implementing the entire HINTS-Exam as a diagnostic tool for patients with AVS. A great advantage is the limited training and knowledge required to perform the exam on the patient, thanks to the voice-guided instructions. Only a brief demonstration was needed for the Head-Impulse-Test. Furthermore, no increase in dizziness was observed during the measurements with the MR-OST-HMD. This is in contrast to what is typically observed when using optical-pass-through HMDs, which often causes cyber-sickness (33, 34).

This is likely due to the OST technology combined with the app’s simplicity and the fact that it relies on voice commands rather than virtual cues. Although the patient group had a representative mean age of 61, a larger sample size is needed to confirm the absence of cyber-sickness and further assess usability (2, 35, 36).

A major strength of the current research is the objective measurements and the device’s portability, allowing non-experts to perform the HINTS-exam and consult at a later stage with qualified personnel. Furthermore, the fact that actual patients are included in this research allows for validation of the application in subsequent research when more patients are included.

The measurement data is used as-is, without artifact correction, noise reduction, or smoothing, as we deemed it unnecessary for this stage and a more honest representation. Other research sites have found the HoloLens 2 sensors to be accurate (37, 38), and this study confirms their usability for eye and head-related measurements. Future research could evaluate accuracy and explore noise reduction techniques with larger datasets.

A current limitation is the lack of automated detection for adequate head impulses during the Head-Impulse-Test with the MR-OST-HMD approach. In our study, experienced clinicians performed this task reliably. However, integrating automatic detection by analyzing the acceleration profile with a minimum threshold of 150°/s (39) and providing audio cues should enhance usability for novice users.

The HL2 is currently priced at 3.500 euros, which is noticeably less than VNG or vHIT equivalents, which are less mobile. Considering that the entire application is built on open-source software, namely Unity and MRTK3, and for post-processing Python, the application and research are replicable and portable to other devices, which should reduce the cost of subsequent research at other research sites.

In conventional nystagmus assessment, Frenzel goggles or analogous instruments are employed to prevent fixation of the eyes, as fixation suppresses nystagmus in patients with peripheral causes of AVS. However, no issues were encountered during this study, and nystagmus was successfully detected in all patients, even in the absence of fixation prevention. However, further data is needed to evaluate if this aspect can be ignored.

### 4.4 Future perspectives

The following steps of our research group are to increase our data volume and apply machine learning.

Before proceeding, we aim to further simplify the application’s usage based on our experience by integrating methods to automatically identify an adequate head impulse, inspect artifact correction and noise reduction, and fine-tune voice instructions and step durations in the MR-OST-HMD HINTS exam.

The aim is to develop a classifier to identify stroke patients and distinguish them from those suffering from peripheral vestibular causes of AVS. The ultimate goal is to develop an algorithm that accurately differentiates peripheral vestibular disorders (harmless) from stroke patients (harmful) without any false negatives. Furthermore, future research should explore the potential of alternative optical-pass-through devices, such as the Meta Quest Pro or Apple Vision Pro (Apple Inc., Cupertino, California, United States), in detecting pathological eye movements and differentiating between the causes of AVS. Specifically, investigations should assess whether the placement of a screen approximately 10 centimeters from the user’s eyes can achieve accurate and reliable measurements.

## 5 Conclusion

The in-house developed MR-OST-HMD HINTS-Exam application showed that the application and device can detect saccades, different types of nystagmus, and test of skew in patients suffering from AVS. The device’s portability and the in-house application’s voice interactivity facilitate straightforward implementation at the point of care. Future research is warranted to gather more data to develop a machine-learning classifier. Hopefully, in the future, it will be possible to improve the triage of patients with AVS using machine learning and HMDs and increase early diagnosis and treatment of stroke patients.

## Code availability

The supplementary materials include a video demonstration of the HINTS exam performed using our in-house developed HoloLens 2 application. To ensure privacy and clarity, the medical personnel’s nose is represented by a black dot, and filming was done from behind.

The Unity and Python code and a recorded sample of a healthy individual will be available on GitHub upon acceptance: https://github.com/TIO-IKIM/AR_HINTS.

## Data Availability

The raw data supporting the conclusions of this article will be made available by the authors, without undue reservation.

## References

[1] Kattah JC Talkad AV Wang DZ Hsieh YH Newman-Toker DE. HINTS to diagnose stroke in the acute vestibular syndrome: three-step bedside oculomotor examination more sensitive than early MRI diffusion-weighted imaging. Stroke. (2009) 40:3504–10. doi: 10.1161/STROKEAHA.109.551234, PMID: 19762709 PMC4593511

[2] Kattah JC. Use of HINTS in the acute vestibular syndrome. An overview. Stroke Vasc Neurol. (2018) 3:190–6. doi: 10.1136/svn-2018-000160, PMID: 30637123 PMC6312070

[3] Newman-Toker DE. Missed stroke in acute vertigo and dizziness: it is time for action, not debate. Ann Neurol. (2016) 79:27–31. doi: 10.1002/ana.24532 26418192 PMC9041814

[4] Kovacs E Wang X Grill E. Economic burden of vertigo: a systematic review. Health Econ Rev. (2019) 9:37. doi: 10.1186/s13561-019-0258-2 31883042 PMC6933936

[5] Comolli L Korda A Zamaro E Wagner F Sauter TC Caversaccio MD. Vestibular syndromes, diagnosis and diagnostic errors in patients with dizziness presenting to the emergency department: a cross-sectional study. BMJ Open. (2023) 13:e064057. doi: 10.1136/bmjopen-2022-064057 PMC10040076 36963793

[6] Choi JH Park MG Choi SY Park KP Baik SK Kim JS. Acute transient vestibular syndrome. Stroke. (2017) 48:556–62. doi: 10.1161/STROKEAHA.116.015507, PMID: 28100765

[7] Tamás TL Garai T Király I Mike A Nagy C Paukovics Á. Emergency diagnosis of the acute vestibular syndrome. Orv Hetil. (2017) 158:2029–40. doi: 10.1556/650.2017.30886, PMID: 29250967

[8] von Werdt M Korda A Zamaro E Wagner F Kompis M Caversaccio MD. The acute vestibular syndrome: prevalence of new hearing loss and its diagnostic value. Eur Arch Otorrinolaringol. (2024) 281:1781–7. doi: 10.1007/s00405-023-08296-z, PMID: 37943315 PMC10942940

[9] Ljunggren M Persson J Salzer J. Dizziness and the acute vestibular syndrome at the emergency department: a population-based descriptive study. Eur Neurol. (2017) 79:5–12. doi: 10.1159/000481982, PMID: 29131011

[10] Nikles F Kerkeni H Zamaro E Korda A Wagner F Sauter TC. Do monosymptomatic stroke patients with dizziness present a vestibular syndrome without nystagmus? An underestimated entity. Eur J Neurol. (2024) 31:e16066. doi: 10.1111/ene.16066, PMID: 37738525 PMC11235630

[11] Tarnutzer AA Berkowitz AL Robinson KA Hsieh YH Newman-Toker DE. Does my dizzy patient have a stroke? A systematic review of bedside diagnosis in acute vestibular syndrome. CMAJ. (2011) 183:E571–92. doi: 10.1503/cmaj.100174, PMID: 21576300 PMC3114934

[12] Kerber KA Brown DL Lisabeth LD Smith MA Morgenstern LB. Stroke among patients with dizziness, vertigo, and imbalance in the emergency department: a population-based study. Stroke. (2006) 37:2484–7. doi: 10.1161/01.STR.0000240329.48263.0d, PMID: 16946161 PMC1779945

[13] Strupp M Bisdorff A Furman J Hornibrook J Jahn K Maire R. Acute unilateral vestibulopathy/vestibular neuritis: Diagnostic criteria. J Vestib Res. (2022) 32:389–406. doi: 10.3233/VES-220201, PMID: 35723133 PMC9661346

[14] Quimby AE Kwok ESH Lelli D Johns P Tse D. Usage of the HINTS exam and neuroimaging in the assessment of peripheral vertigo in the emergency department. J Otolaryngol Head Neck Surg. (2018) 47:54. doi: 10.1186/s40463-018-0305-8, PMID: 30201056 PMC6131950

[15] Newman-Toker DE Kerber KA Hsieh YH Pula JH Omron R Saber Tehrani AS. HINTS outperforms ABCD2 to screen for stroke in acute continuous vertigo and dizziness. Acad Emerg Med. (2013) 20:986–96. doi: 10.1111/acem.12223, PMID: 24127701

[16] Nham B Akdal G Young AS Özçelik P Tanrıverdizade T Ala RT. Capturing nystagmus in the emergency room: posterior circulation stroke versus acute vestibular neuritis. J Neurol. (2023) 270:632–41. doi: 10.1007/s00415-022-11202-y, PMID: 35849153 PMC9886594

[17] Rayner R Hartley-Palmer J Hmu C. Use of the head impulse, nystagmus, test of skew (‘HINTS’) assessment to aid differential diagnosis in acute vestibular syndrome in the hyperacute stroke setting. J Laryngol Otol. (2024) 138:S14–7. doi: 10.1017/S0022215123002050, PMID: 38779899

[18] Zhang X Deng Q Liu Y Li S Wen C Liu Q. Characteristics of spontaneous nystagmus and its correlation to video head impulse test findings in vestibular neuritis. Front Neurosci. (2023) 17:1243720. doi: 10.3389/fnins.2023.1243720, PMID: 37674516 PMC10477358

[19] Walther LE. Current diagnostic procedures for diagnosing vertigo and dizziness. GMS Curr Top Otorhinolaryngol Head Neck Surg. (2017) 16:Doc02. doi: 10.3205/cto000141 29279722 PMC5738933

[20] Luengo-Fernandez R Li L Silver L Gutnikov S Beddows NC Rothwell PM. Long-term impact of urgent secondary prevention after transient ischemic attack and minor stroke: ten-year follow-up of the EXPRESS study. Stroke. (2022) 53:488–96. doi: 10.1161/STROKEAHA.121.034279, PMID: 34706563 PMC8785519

[21] Mixed Reality Toolkit 3. Developer Documentation - MRTK3. (2023). Available online at: https://learn.microsoft.com/en-us/windows/mixed-reality/mrtk-unity/mrtk3-overview/ (Accessed March 31, 2025).

[22] Extended eye tracking in Unity. Mixed reality. (2022). Available online at: https://learn.microsoft.com/en-us/windows/mixed-reality/develop/unity/extended-eye-tracking-unity (Accessed March 31, 2025).

[23] The Python Software Foundation. Python. Wilmington, Delaware, United States. (2023). Available online at: https://python.org (Accessed March 31, 2025).

[24] The Pandas Development Team. Pandas. (2023). Available online at: https://pandas.pydata.org/ (Accessed March 31, 2025).

[25] Plotly Technologies Inc. Plotly. (2023). Available online at: https://plotly.com/python/ (Accessed March 31, 2025).

[26] Gsaxner C Li J Pepe A Jin Y Kleesiek J Schmalstieg D. The HoloLens in medicine: a systematic review and taxonomy. Med Image Anal. (2023) 85:102757. doi: 10.1016/j.media.2023.102757, PMID: 36706637

[27] Palumbo A. Microsoft HoloLens 2 in medical and healthcare context: state of the art and future prospects. Sensors. (2022) 22:7709. doi: 10.3390/s22207709, PMID: 36298059 PMC9611914

[28] Margani V Pascucci S Talamonti R Serani E Bini F Marinozzi F. Augmented virtual reality in vestibular assessment: a dynamic gait application. Audiol Neurootol. (2023) 28:308–16. doi: 10.1159/000529993, PMID: 37071980

[29] González-Vides L Hernández-Verdejo JL Cañadas-Suárez P. Eye tracking in optometry: a systematic review. J Eye Mov Res. (2023) 16. doi: 10.16910/jemr.16.3.3, PMID: 38111688 PMC10725735

[30] Cho C Park S Ma S Lee HJ Lim EC Hong SK. Feasibility of video-based real-time nystagmus tracking: a lightweight deep learning model approach using ocular object segmentation. Front Neurol. (2024) 15:1342108. doi: 10.3389/fneur.2024.1342108, PMID: 38450068 PMC10915048

[31] Kukushev G Yanev D Pramatarov A. Videonystagmography to go. Int Bull Otorhinolaryngol. (2023) 19:15. doi: 10.14748/orl.v19i3.9773

[32] Kuroda T Kuroda K Fushiki H. Development of a prototype video head impulse test system using an iPhone for screening of peripheral vestibular dysfunction. Digit Biomark. (2023) 7:150–6. doi: 10.1159/000534543, PMID: 37928503 PMC10622167

[33] Tian N Lopes P Boulic R. A review of cybersickness in head-mounted displays: raising attention to individual susceptibility. Virtual Reality. (2022) 26:1409–41. doi: 10.1007/s10055-022-00638-2

[34] Kirollos R Merchant W. Comparing cybersickness in virtual reality and mixed reality head-mounted displays. Front Virtual Real. (2023) 4:4. doi: 10.3389/frvir.2023.1130864

[35] Ohle R Montpellier RA Marchadier V Wharton A McIsaac S Anderson M. Can emergency physicians accurately rule out a central cause of vertigo using the HINTS examination? A systematic review and Meta-analysis. Acad Emerg Med. (2020) 27:887–96. doi: 10.1111/acem.13960, PMID: 32167642

[36] Gottlieb M Peksa GD Carlson JN. Head impulse, nystagmus, and test of skew examination for diagnosing central causes of acute vestibular syndrome - Gottlieb, M - 2023 | Cochrane Library. (2025). Available online at: https://www.cochranelibrary.com/cdsr/doi/10.1002/14651858.CD015089.pub2/abstract?cookiesEnabled (Accessed March 31, 2025). 10.1002/14651858.CD015089.pub2 PMC10620998 37916744

[37] Balakrishnan P Guo HJ. HoloLens 2 technical evaluation as mixed reality guide In: Chen JYC Fragomeni G, editors. Virtual, augmented and mixed reality. Cham: Springer Nature Switzerland (2024). 145–65.

[38] Kapp S Barz M Mukhametov S Sonntag D Kuhn J. ARETT: augmented reality eye tracking Toolkit for head mounted displays. Sensors. (2021) 21:2234. doi: 10.3390/s21062234 33806863 PMC8004990

[39] LA MG HG MD Halmagyi GM Burgess AM Weber KP Curthoys IS. The video head impulse test (vHIT) of semicircular canal function–age-dependent normative values of VOR gain in healthy subjects. Front Neurol. (2015) 6:154. doi: 10.3389/fneur.2015.00154 26217301 PMC4495346

